# Effects of Perilla Seed Pomace Extract on Aging-Induced Cognitive Dysfunction in SAMP8 Mice

**DOI:** 10.4014/jmb.2502.02007

**Published:** 2025-06-12

**Authors:** Haeun Lee, Tae Youl Ha, Yun Tai Kim, Min Young Um

**Affiliations:** 1 Food Functionality Research Division, Korea Food Research Institute, Wanju 55365, Republic of Korea; 2 Department of Food Biotechnology, University of Science & Technology, Daejeon 34113, Republic of Korea

**Keywords:** Perilla seed, antioxidant, apoptosis, aging, cognitive deficits

## Abstract

Perilla seed pomace and its constituent phytochemicals have anti-inflammatory, antioxidant, and anti-aging benefits. However, its effects on aging-related cognitive decline are not well-studied. Therefore, the antioxidant and neuroprotective properties of perilla seed pomace extract (PSE) on hydrogen peroxide (H_2_O_2_)-induced neuronal cell death were examined. Additionally, the efficacy of PSE in ameliorating age-related cognitive impairment was evaluated *in vivo* using the senescence-accelerated mouse prone 8 (SAMP8). PSE showed free radical scavenging activities and inhibited FeSO_4_-H_2_O_2_-induced lipid peroxidation. In HT22 neuronal cells, PSE protected against H_2_O_2_-induced oxidative stress and cytotoxicity by restoring mitochondrial membrane potential and attenuating excessive reactive oxygen species production. Furthermore, PSE reversed the H_2_O_2_-induced upregulation of apoptotic markers (Bax, cleaved caspase-3) and downregulation of the anti-apoptotic marker Bcl-2. Additionally, the protein levels of antioxidant enzymes (Catalase, SOD, and GPx) were increased under conditions of H_2_O_2_-induced oxidative stress. *In vivo*, PSE significantly ameliorated cognitive deficits, as evidenced by improved performance in passive avoidance and Morris water maze test results. Further, PSE reduced the accumulation of lipid peroxides and significantly increased catalase and glutathione peroxidase activity in the brain. These findings suggest that PSE attenuates aging-related cognitive deficits by enhancing antioxidant defenses in the brain, highlighting its potential as a dietary intervention for aging-related cognitive decline.

## Introduction

As the global population ages, neurodegenerative disorders have become a critical public health concern. Among environmental factors, oxidative stress induced by free radicals serves a central role in aging-related physiological decline [1]. Oxidative stress is involved in the pathogenesis of various neurodegenerative diseases including Alzheimer’s disease (AD) [2], with excessive oxidative damage to biomolecules (*e.g.*, lipids and proteins) being a hallmark of these diseases [3]. As such, developing strategies to mitigate oxidative stress and its deleterious effects is important. Dietary antioxidants have emerged as a promising approach for counteracting oxidative stress, with the potential to preserve cognitive function and delay disease progression [4].

Perilla seeds have been traditionally used in Asian culinary and medicinal practices. Perilla seed pomace, a by-product of oil extraction, is commonly discarded or utilized as a low-cost protein source in animal feed [5]. However, recent studies have identified health-beneficial bioactive compounds in perilla seed pomace, revealing its potential as a valuable resource [6]. These include flavonoids (apigenin, luteolin) and phenolic acids (rosmarinic acid), which have been recognized as key components contributing to its antioxidant properties [7, 8]. Also, these phytochemicals have various biological properties, anti-inflammatory and anti-aging benefits [6, 9, 10]. However, research on the effect of perilla seed pomace on aging-related cognitive decline remains limited.

Therefore, this study investigated the neuroprotective potential of perilla seed pomace extract (PSE). To assess the antioxidant and neuroprotective properties of PSE, we evaluated its effects on hydrogen peroxide (H_2_O_2_)-induced neuronal cell death. Additionally, its antioxidant capacity observed using cell-free *in vitro* assays. Finally, we examined the efficacy of PSE in ameliorating aging-related cognitive impairment using the SAMP8 mouse model, a well-established system for studying brain aging.

## Materials and Methods

### Perilla Seed Pomace Extract Preparation

PSE, the residue remaining after oil extraction by pressing, was obtained from Kyungdong Oriental Market (Republic of Korea). To prepare the PSE, the perilla seed pomace was soaked in n-hexane to remove any residual oil. The defatted pomace was extracted with 60% ethanol in a ratio of 1:10 (w/v). The ethanolic extract was filtered, concentrated under reduced pressure using a rotary vacuum evaporator (Büchi R-114, Switzerland), freeze dried, and stored at -80°C. The total polyphenol contents of PSE were 14.45 ± 0.59 mg tannic acid equivalent per gram of extract.

### 2,2'-Azino-Bis-(3-Ethylbenzo-Thiazoline-6-Sulfonic Acid) Radical Scavenging Assay

2,2'-Azino-bis-(3-ethylbenzo-thiazoline-6-sulfonic acid) (ABTS) radical scavenging activity was measured using an antioxidant assay kit (Sigma-Aldrich, USA). Absorbance at 405 nm was measured using a microplate reader (BioTek Epoch, USA).

### 2,2-Diphenyl-1-Picrylhydrazyl Radical Scavenging Assay

The 2,2-diphenyl-1-picrylhydrazyl (DPPH) free radical scavenging assay was conducted as previously described [11]. Absorbance was measured at 517 nm using a microplate reader (BioTek Epoch).

### Measurement of Thiobarbituric Acid Reactive Substance Levels

Thiobarbituric acid reactive substance (TBARS) levels were measured with slight modifications as previously described [12]. For the *in vitro* experiments, homogenates of rat forebrain tissue (1:10, w/v) were freshly prepared in ice-cold 20 mM Tris-HCl buffer (pH 7.4) using a Qsonica homogenizer (Qsonica LLC, USA). Lipid peroxidation was induced by incubating 0.2 ml of the rat brain homogenate with 10 mM FeSO_4_ (0.1 ml), 0.5% H_2_O_2_ (0.1 ml), and samples (20 μl) at 37°C for 20 min. Following incubation, 0.2 ml 8.1% sodium dodecyl sulfate (SDS), 1.5 ml 0.8%thiobarbituric acid, and 1.5 ml 20% acetic acid (adjusted to pH 3.5 with NaOH) were added to the mixture. The mixture was then boiled at 95°C for 40 min. After cooling on ice, precipitated proteins were removed by centrifugation at 1,500 ×g for 15 min. Absorbance was measured at 532 nm using a microplate reader (BioTek Epoch). The lipid peroxidation inhibitory activity was expressed as the percentage of sample absorbance relative to the control, after blank correction. For *in vivo* experiments, lipid peroxidation in the brains of mice was assessed by measuring TBARS production. Following the same TBARS assay protocol, TBARS levels were expressed as nanomoles per gram of brain tissue (nmol/g brain tissue) using a standard malondialdehyde (MDA) curve.

### Cell Culture and Viability

HT22 mouse hippocampal neuronal cells were cultured in DMEM supplemented with 10% FBS and 100 U/ml penicillin-streptomycin. The protective effects of PSE on cell viability in H_2_O_2_-treated cells were measured using the thiazolyl blue tetrazolium bromide (MTT) assay. The cells were seeded in 96-well plates at a density of 1.0 × 10^4^ cells/well. After 24 h, the cells were treated with 750 μM H_2_O_2_, with or without PSE, for 12 h. MTT solution was added and incubated for 3 h, followed by addition of 150 μL DMSO. Absorbance was read at 570 nm using a microplate reader (BioTek Epoch).

### Measurement of Apoptosis

Apoptotic cell and the morphology was assessed through an Annexin V/FITC and propidium iodide (PI) staining kit (Abcam, UK) and Hoechst staining (Invitrogen). For Annexin V/FITC and PI staining, cells were seeded in a 6-well plate at a density of 5 × 10^5^ cells/well and allowed to adhere for 24 h. Subsequently, the cells were treated with 750 μM H_2_O_2_, with or without various concentrations of PSE, for 12 h. After treatment, the cells were harvested by trypsinization, followed by centrifugation for 3 min. They were stained with Annexin V/FITC and PI for 15 min in the dark. Apoptotic cells were analyzed using a Beckman flow cytometer (Beckman Coulter, USA).

For Hoechst staining, the cells were seeded in a 96-well plate at a density of 4 × 10^3^ cells/well and allowed to adhere for 24 h. They were then treated with 750 μM H_2_O_2_, with or without PSE, for 12 h. Thereafter, cells were incubated with Hoechst dye at 37°C for 10 min under dark conditions. After washing with HBSS, apoptotic nuclear morphology was assessed using confocal microscopy (Zeiss, Germany).

### Measurement of Mitochondrial Membrane Potential

Mitochondrial membrane potential (MMP) was assessed by JC-1 staining. Cells were seeded in a 96-well plate and treated with 750 μM H_2_O_2_, with or without PSE, for 12 h. Following treatment, cells were incubated with JC-1 dye at 37°C for 30 min under dark conditions. Subsequently, the dye was removed, and the samples were imaged using a confocal microscope. MMP was quantified by calculating the ratio of red to green fluorescence in the captured images.

### Measurement of Reactive Oxygen Species Production

The production of intracellular reactive oxygen species (ROS) was measured using the 2’−7’-dichloro-dihydrofluorescein diacetate (DCF-DA) assay. ROS levels were determined by capturing fluorescence images using a microscope and were quantified by measuring the absorbance at 485/538 nm using a microplate reader (BioTek Epoch).

### Immunoblotting

The cells were lysed in RIPA buffer containing phosphatase inhibitors, and lysates were centrifuged (13,000 × *g*, 10 min, 4°C) to collect the supernatants. Protein concentration was determined using a BCA assay kit. Samples were mixed with SDS sample buffer, denatured at 95°C for 5 min, and separated on 10–12% SDS-PAGE. Proteins were transferred to PVDF membranes and blocked with 5% skim milk in TBST. Subsequently, membranes were then incubated overnight at 4°C with primary antibodies against β-actin, caspase-3, Bcl-2, cleaved caspase-3, Bax, superoxide dismutase (SOD), glutathione peroxidase 1 (GPx1) (all form Cell Signaling Technology (CST), USA), and catalase (Abcam). After washing, membranes were incubated with HRP-linked secondary antibodies for 1 h at room temperature. Protein bands were visualized using a chemiluminescence imaging system (LuminoGraph II EM, ATTO, Japan) and quantified using ImageJ software (NIH, USA).

### Animals and Experimental Design

All experimental protocols were approved by the Institutional Animal Care and Use Committee of the Korea Food Research Institute (approval number: KFRI-M-23054). Twelve-week-old male senescence-accelerated mouse prone 8 (SAMP8) mice were obtained from Central Lab. Animal Inc. (Republic of Korea). The mice were housed under a 12-h light-dark cycle at a constant temperature of 23-25°C and humidity of 55 ± 5% until the reached 9 months of age. They were divided into 3 groups and provided a specific diet for 12 weeks: (1) CON group: SAMP8 mice fed AIN-76 diet (*n* = 10); (2) PSE 0.25% group: SAMP8 mice fed AIN-76 diet supplemented with 0.25% PSE (*n* = 10); and (3) PSE 0.5% group: SAMP8 mice fed AIN-76 diet supplemented with 0.5% PSE (*n* = 10). The PSE dose was selected with reference to previous study and literature [13, 14]. The composition of the experimental diet is presented in Table 1. After the experimental period, behavioral tests were conducted, and the mice were sacrificed. Serum and brain tissues were collected and stored at -80°C.

### Behavioral Tests

**Passive avoidance test.** Passive avoidance test (PAT) was conducted as previously described [15, 16]. The experimental setup utilized a GEMINI chamber (San Diego Instruments, USA) that consisted of two compartments (a dark chamber and a light chamber). During the training phase, each mouse was placed in the light chamber. Upon entry into the dark chamber, mice received an electric foot shock (0.3 mA) for 3 sec. The test trial was conducted the following day. Each mouse was again placed in the light chamber, and the latency to enter the dark chamber was recorded. Latency was defined as the time from door opening to entry into the dark chamber, with a 300-sec cutoff.

**Morris water maze test.** The Morris water maze test (MWMT) was performed with slight modifications from the previous Morris water task protocol [17]. The test consisted of training and probe trial phases. During the 4-day training phase, the mice were trained to locate the submerged platform within a set time frame (120 sec). If a mouse failed to locate the platform, it was placed on it for 30 sec to aid spatial learning. On the fifth day, the platform was removed, and the time taken by each mouse to reach the platform’s previous location (escape latency) and the distance they swam (swimming path length) were measured using a video tracking system (Panlab, Spain).

### Measurement of Antioxidant Enzyme Activity

Whole brain tissues were homogenized in 10 volumes (w/v) of buffer containing 0.25 M sucrose and 0.5 mM EDTA, and the homogenate was centrifuged at 1,000 × *g* for 5 min at 4°C. An aliquot of the supernatant was collected for catalase activity assay. For SOD and GPx assays, the supernatant was further centrifuged at 10,000 ×g for 30 min at 4°C. Catalase activity was measured as described by Aebi [18]. SOD activity was assessed using the method of Marklund and Marklund [19]. GPx activity was measured as previously described by Lawrence and Burk [20]. Protein concentration was determined using the method described by Lowry [21] with bovine serum albumin as a standard.

### Statistical Analysis

All data were presented as mean ± standard error of the mean, and differences were analyzed by one-way analysis of variance (ANOVA), followed by Tukey’s post hoc test. All statistical analyses were performed using Prism 10 (GraphPad Software, Inc., USA). Statistical significance was set at *P* < 0.05.

## Results

### Antioxidant Activity of PSE

**Fig. 1:**
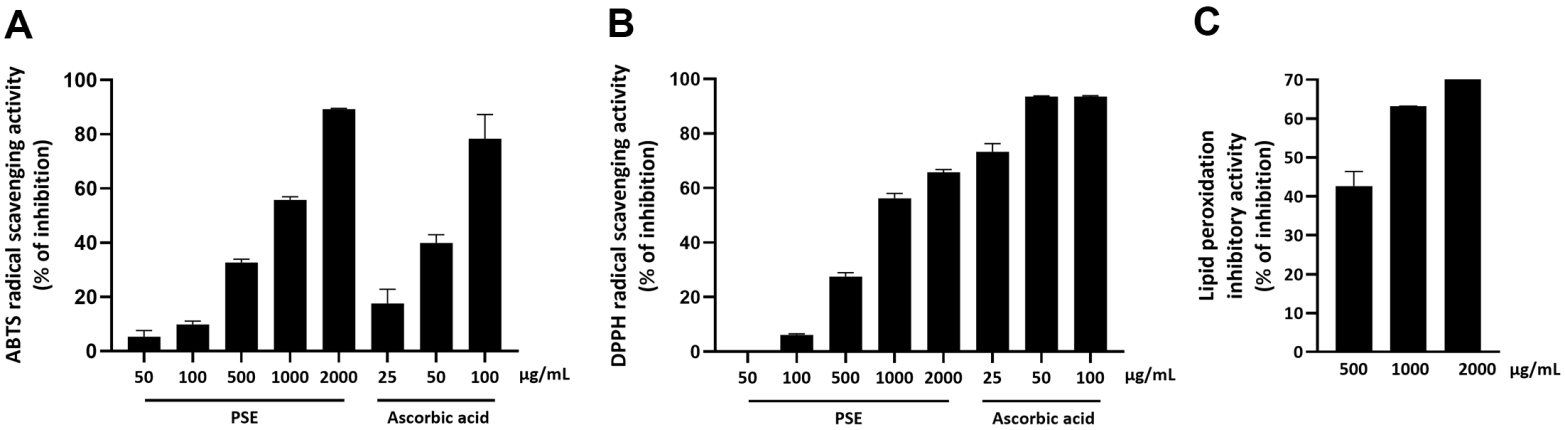
Antioxidant activity of PSE. (**A**) DPPH scavenging activity, (**B**) ABTS radical scavenging activity, and (**C**) inhibitory activity of lipid peroxidation. Data are expressed as the means ± standard error from three independent experiments.

The radical scavenging activities of PSE against both DPPH and ABTS increased in a dose-dependent manner (Fig. 1A and 1B). The IC50 values for PSE were 995 ± 71.74 μg/mL for DPPH and 771.37 ± 40.16 μg/ml for ABTS. In the TBARS assay, PSE significantly reduced the MDA levels in a dose-dependent manner, demonstrating its ability to inhibit lipid peroxidation (Fig. 1C). These results demonstrate that PSE exhibits antioxidant properties.

### Effect of PSE on Apoptosis in H_2_O_2_-Treated HT22 Cells

**Fig. 2:**
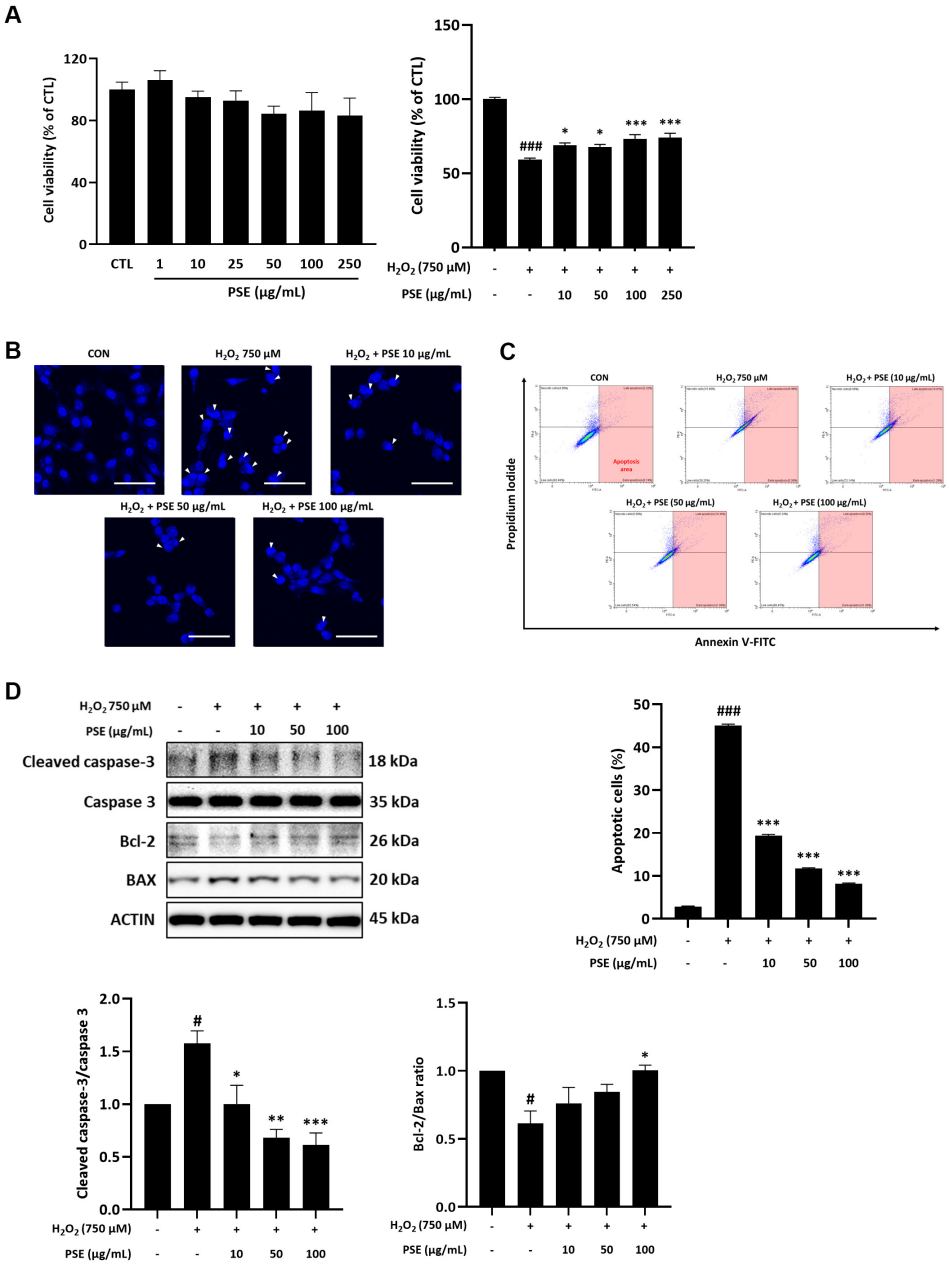
Protective effects of PSE against H_2_O_2_-induced cytotoxicity and apoptosis in HT22 cells. (**A**) Cell viability, (**B**) Hoechst staining (magnification: 60×; scale bar: 100 μM), and (**C**) Annexin V/FITC and PI staining. Arrows represent apoptotic cells. Representative dot plots (2,000 dots shown from a total of 20,000 analyzed dots) and the corresponding quantitative graph showing the percentage of apoptotic cells are presented. (**D**) Cleaved caspase-3, Bax, and Bcl- 2 protein levels. Data are obtained from three independent experiments. ^#^*P* < 0.05 and ^###^*P* < 0.001 compared with the CON group; *P < 0.05, **P < 0.01, and ***P < 0.001 compared with the H_2_O_2_ group

Exposure to 750 μM H_2_O_2_ for 12 h resulted in a dose-dependent reduction in cell viability (data not shown). Specifically, cell viability decreased by 48.66 ± 5% following treatment with 750 μM H_2_O_2_ (*P* < 0.001). Additionally, when PSE was treated alone at concentrations ranging from 1 to 250 μg/ml for 12 h, cell viability not showed cytotoxic effect at concentrations by 250 μg/ml. Based on these findings, we treated the cells with 750 μM H_2_O_2_ in the presence of varying concentrations of PSE for 12 h. PSE significantly increased cell viability, which was reduced by H_2_O_2_ treatment (Fig. 2A). On Hoechst staining, the nuclei in the H_2_O_2_ only-treated cells exhibited condensation, and this was notably alleviated by PSE treatment (Fig. 2B). Also, Annexin V/FITC and PI staining analyzed by flow cytometry further confirmed a significant reduction in the number of apoptotic cells by H_2_O_2_ after PSE exposure (Fig. 2C). Western blotting analysis further showed that H_2_O_2_ significantly increased cleaved caspase-3 and Bax protein levels, whereas it increased Bcl-2 protein levels. These findings suggest that PSE decreases apoptosis in H_2_O_2_-damaged HT22 cells. However, these changes were significantly reversed by PSE treatment, indicating that PSE had a protective effect against apoptosis.

### Effect of PSE on Oxidative Damage in H_2_O_2_-Treated HT22 Cells

**Fig. 3:**
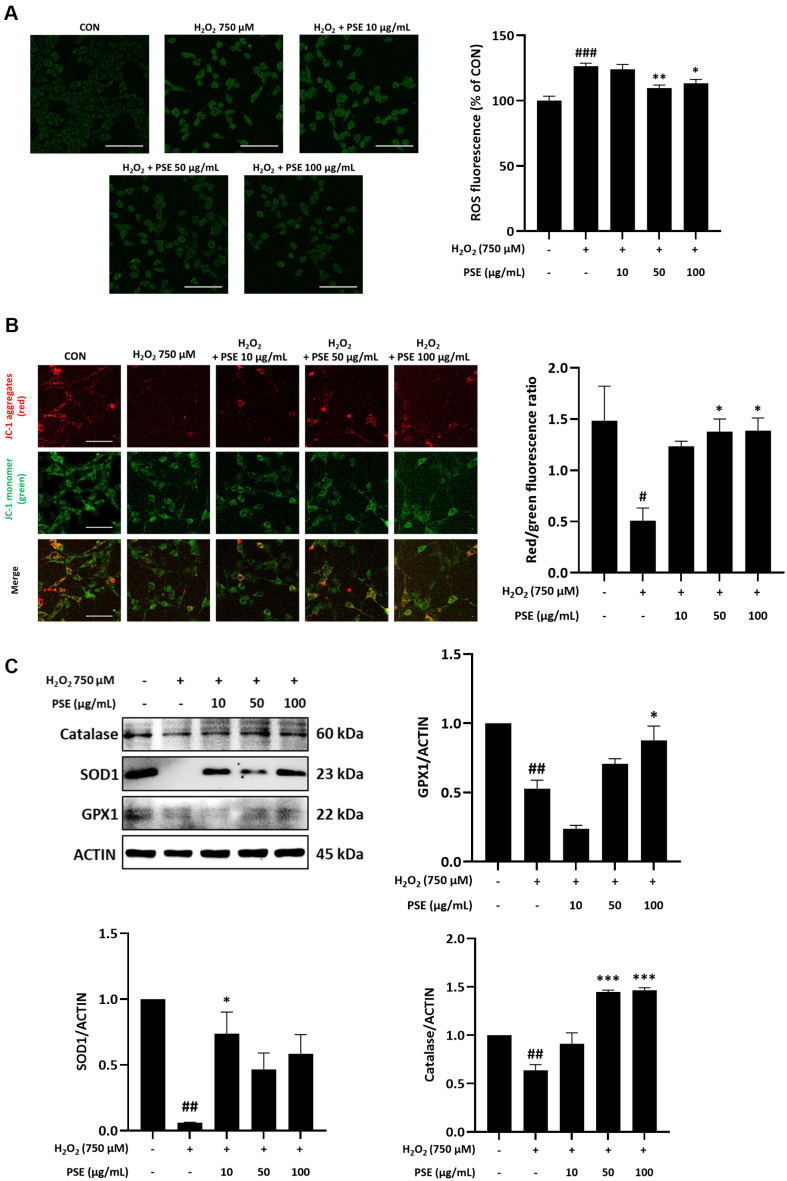
Effect of PSE on H_2_O_2_-induced oxidative stress in HT22 cells. (**A**) ROS levels quantified by DCF-DA fluorescence analysis. (**B**) Mitochondrial membrane potential assessed using JC-1 staining, showing changes in the red/green fluorescence ratio. (**C**) The protein levels of GPx1, SOD1, and catalase. Data are obtained from three independent experiments. ^#^*P* < 0.05, ^##^*P* < 0.01, and ^###^*P* < 0.001 compared with the CON group; *P < 0.05, **P < 0.01, and ***P < 0.001 compared with the H_2_O_2_ group.

In DCF-DA staining, H_2_O_2_ exposure significantly increased ROS production, with levels notably higher than those in the CON group (Fig. 3A). However, PSE treatment significantly inhibited the H_2_O_2_-induced elevation of intracellular ROS levels. JC-1 staining images revealed aggregated forms (red fluorescence) in the matrix of normal mitochondria. In contrast, monomer forms (green fluorescence), which indicated mitochondrial membrane damage caused by cell death. Compared with the H_2_O_2_ only-treated group, the PSE-treated group showed higher red/green fluorescence ratio (Fig. 3B), indicating restoration of MMP. Additionally, western blot analysis revealed decreased expression of antioxidant enzymes, including GPx1, catalase, and notably SOD1, following H_2_O_2_ treatment. However, these changes were reversed upon treatment with PSE (Fig. 3C). Collectively, these results demonstrate that PSE can effectively alleviate oxidative damage.

### Effect of PSE on Cognitive Dysfunction and Oxidative Stress

**Fig. 4:**
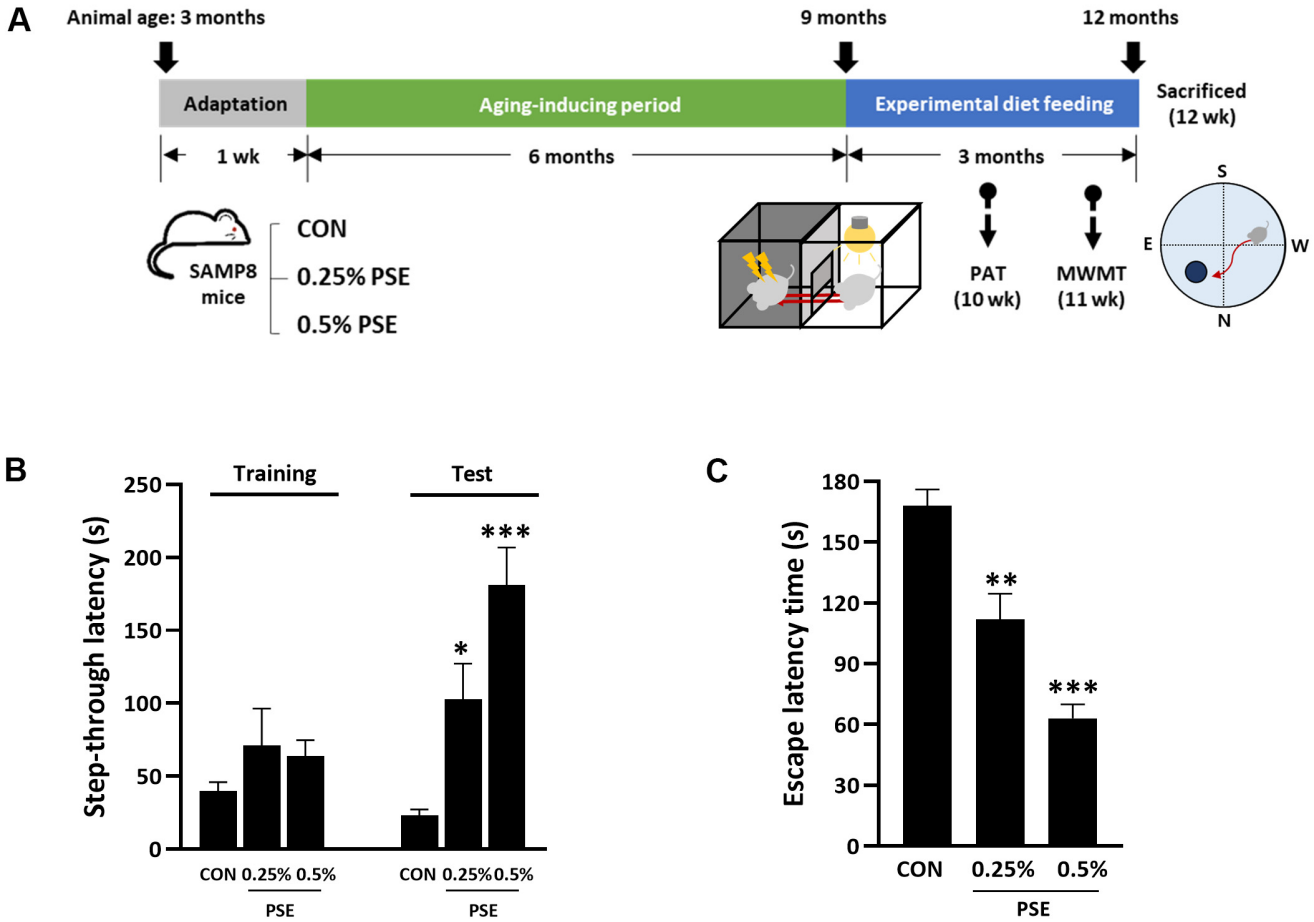
PSE ameliorates cognitive dysfunction in SAMP8 mice. (**A**) Experimental design and schedule. The mice receive an AIN-76 diet containing 0.25% or 0.5% PSE starting at 9 months of age for 12 week, and subjected to behavioral tests. (**B**) Step-through latency (s) in the PAT. (**C**) Escape latency on the final test day (day5) in the MWMT. Data are expressed as the means ± standard error. *P < 0.05, **P < 0.01, and ***P < 0.001 compared with the CON group.

The experimental schedule, including the PSE supplementation period and assessment time points, is shown in Fig. 4A. In the PAT, the step-through latency of the CON group reduced by 23 sec, whereas that of the PSE-administrated groups significantly increased, with the values being 102 sec and 180 sec in the 0.25% PSE and 0.5%PSE groups (*P* < 0.05 and *P* < 0.001, respectively). Particularly, step-through latency was 9-fold higher in the 0.5%PSE group than that in the CON group (Fig. 4B). In the MWMT, escape latency in the PSE-administered groups was significantly shorter than that in the CON group (Fig. 4C). These results suggest that PSE administration effectively improves cognitive function in SAMP8 mice.

With respect to the lipid peroxide levels and antioxidant enzyme activities in the brain tissues of SAMP8 mice, PSE administration significantly decreased the TBARS levels in the 0.5% PSE group (*P* < 0.05, Table 2). Catalase activity in the 0.5% PSE group was also significantly increased (*P* < 0.05). Interestingly, GPx activity was 50%higher in the 0.5% PSE group than that in the CON group. However, PSE administration did not affect SOD activity in the brain. Collectively, these results suggested that the cognitive improvements observed following PSE administration may be attributed to its antioxidant properties.

## Discussion

In the present study, we investigated the neuroprotective effects of PSE mediated by its antioxidant properties using HT22 neuronal cells. These effects were evaluated by assessing MMP, ROS and apoptosis-related markers. Moreover, the antioxidant potential of PSE was confirmed *in vivo* using SAMP8 mouse model by measuring antioxidant enzyme activity and lipid peroxidation levels. Ultimately, the neuroprotective effects of PSE, driven by its antioxidant properties, were shown to enhance cognitive function through behavioral tests in mice.

Oxidative stress generally leads to the excessive generation and accumulation of ROS, resulting in MMP disruption [22]. The interaction between elevated ROS levels and MMP disruption forms a feedback loop that accelerates apoptosis. The apoptosis pathway influenced by this loop is driven by a mitochondria-dependent mechanism that activates the caspase cascade [23, 24]. Key regulators include members of the Bcl-2 family, which modulate the antioxidant system to determine cell survival or death. The ratio of pro-apoptotic proteins (Bax) to anti-apoptotic proteins (Bcl-2) plays a critical role in downstream caspase activation [25]. As previously reported [26, 27], H_2_O_2_ treatment reduces Bcl-2 protein levels and elevates Bax protein levels, ultimately increasing the expression of cleaved caspase-3, the active form that leads to apoptotic cell death. Consistent with this mechanism, our data showed that H_2_O_2_ exposure in neuronal cells elevated ROS levels and caused MMP disruption. These changes were associated with induction of apoptosis and cell death. However, PSE effectively inhibited H_2_O_2_-induced caspase-3 activation and restored the Bax/Bcl-2 ratio to control levels. Collectively, these findings suggest that PSE modulates the functional and apoptotic changes induced by oxidative stress, thereby protecting neuronal cells.

Next, we investigated whether the antioxidant and neuroprotective effects of PSE manifested similarly in an SAMP8 mouse model. Previous studies [28-30] have shown that SAMP8 mice exhibit higher oxidative stress, increased lipid peroxidation, and impaired cognitive function compared to SAMR1 mice. Behavioral experiment results showed aging-related cognitive decline in SAMP8, which was ameliorated by PSE supplementation, as evidenced by increased step-through latency in the PAT and decreased escape latency in the MWMT. Additionally, TBARS levels in the brain were significantly reduced, indicating decreased lipid peroxidation. GPx and catalase, key enzymes involved in neutralizing H_2_O_2_, played a crucial role in this process [31]. Although SOD activity remained unchanged in the PSE-treated group, the increased activity of GPx and catalase demonstrated that PSE effectively enhanced the antioxidant defense system. This reduction in lipid peroxidation and antioxidant enzymatic changes can be attributed to bioactive compounds, such as flavonoids, omega-3 fatty acids, and phenolic acids found in perilla seeds [32-34], suggesting a potential role for perilla-derived antioxidants in cognitive function.

Although these findings demonstrate promising outcomes with respect to improved cognitive performance and enhanced antioxidant defenses in the SAMP8 mouse model, some limitations must be acknowledged. The specific bioactive compounds responsible for the observed effects of PSE are yet to be identified. Further research is required to isolate and characterize these compounds. Although this study provides important foundational data, the efficacy and safety of PSE still need to be confirmed in well-designed human clinical trials.

In conclusion, PSE exerts significant neuroprotective and antioxidant effects, alleviates oxidative stress, and improves cognitive function in both cellular and animal models. These findings highlight the potential of PSE as a dietary intervention for aging-related cognitive decline and neurodegenerative disorders.

## Figures and Tables

**Table 1:** Composition of experimental diets (g/kg diet)

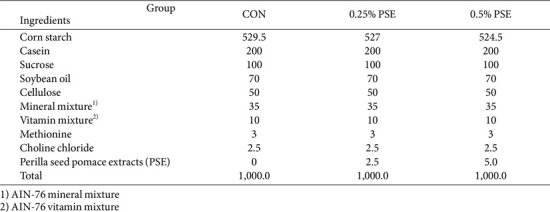

**Table 2:** Lipid peroxide and antioxidant enzyme activities of catalase, SOD, and GPx in the brain of SAMP8 mice.

	Catalase (units/min/mg protein)	SOD (units/min/mg protein)	GPx (nmol/min/mg protein)	TBARS (MDA nmol/g brain tissue)
CON	3.4 ± 0.2^1)^	3.7 ± 0.2	28.3 ± 2.4	54.0 ± 0.7
0.25% PSE	3.4 ± 0.2	3.7 ± 0.1	41.9 ± 2.2^*^	52.2 ± 0.6
0.5% PSE	4.0 ± 0.1^*,2)^	3.6 ± 0.1	48.8 ± 9.2^*^	50.4 ± 0.6^*^

1) Values were mean ± standard error (*n* = 10 per group)

2) * *P* < 0.05, compared with CON group
